# Moral Chivalry: Gender and Harm Sensitivity Predict Costly Altruism

**DOI:** 10.1177/1948550616647448

**Published:** 2016-05-25

**Authors:** Oriel FeldmanHall, Tim Dalgleish, Davy Evans, Lauren Navrady, Ellen Tedeschi, Dean Mobbs

**Affiliations:** 1 Department of Psychology, New York University, New York, NY, USA; 2 Medical Research Council, Cognition and Brain Sciences Unit, Cambridge, UK; 3 School of Psychology, University of Birmingham, Birmingham, UK; 4 Department of Psychology, Columbia University, New York, NY, USA

**Keywords:** moral, altruism, gender, gender bias, harm sensitivity

## Abstract

Moral perceptions of harm and fairness are instrumental in guiding how an individual navigates moral challenges. Classic research documents that the gender of a target can affect how people deploy these perceptions of harm and fairness. Across multiple studies, we explore the effect of an individual’s moral orientations (their considerations of harm and justice) and a target’s gender on altruistic behavior. Results reveal that a target’s gender can bias one’s readiness to engage in harmful actions and that a decider’s considerations of harm—but not fairness concerns—modulate costly altruism. Together, these data illustrate that moral choices are conditional on the social nature of the moral dyad: Even under the same moral constraints, a target’s gender and a decider’s gender can shift an individual’s choice to be more or less altruistic, suggesting that gender bias and harm considerations play a significant role in moral cognition.

## Introduction

A culturally pervasive social norm is the chivalrous idea that women should be protected from harm. This is exemplified by “women and children first”—a historical maritime code of conduct stating that when there is a life-threatening situation, those who are more vulnerable should be saved first (Kipling, 1907). Dovetailing with this, classic research on gender stereotyping demonstrates that both implicit judgments (Banaji & Hardin, 1996a) and explicit actions (Eagly & Crowley, 1986; Moss-Racusin, Dovidio, Brescoll, Graham, & Handelsman, 2012) are affected by gender bias—that is, associating males with strength and power and females with nurturance and helplessness. This reliance on using a target’s features to infer overarching personality traits (Asch, 1946) has proven to be powerful in influencing one’s judgments (Hamilton & Sherman, 1996; Higgins, Rholes, & Jones, 1977) and even behavior (Tversky & Kahneman, 1981).

From this work, theorists have further posited that a target’s features may affect endorsements of harm violation (Gray, Waytz, & Young, 2012), which fits with the evidence that harming behavior in particular is susceptible to shifting social cues that signal distinct morally appropriate behavior. Research exploring the interaction between harming and helping demonstrates that how readily an individual harms another appears to be a function of the social context in which the harm is embedded. For example, features that make harm perceptually salient decrease the likelihood of engaging in harmful behavior. This has been observed in various classes of moral dilemmas and with different manipulations, including pushing a person onto train tracks versus pulling a lever to reroute the train (Greene, Sommerville, Nystrom, Darley, & Cohen, 2001), showing more versus less skin when administering electric shocks (Gray, Knobe, Sheskin, Bloom, & Barrett, 2011), observing someone’s face rather than just their hand respond to pain (FeldmanHall, Dalgleish, Evans, & Mobbs, 2015), and discharging a toy gun into another’s face versus witnessing such an action occurring (Cushman, Gray, Gaffey, & Mendes, 2012).

Even after the harm has occurred, social context can further influence how one engages with a distressed target. For instance, a target’s identity is known to effect the level of sympathy or punishment that is bestowed (Cikara, Bruneau, & Saxe, 2011; Gray & Wegner, 2009), and how a person responds to a target’s pain is moderated by their relationship with the distressed individual (Cikara, Botvinick, & Fiske, 2011). Given how robustly social context can dictate harm perception, a lingering question is whether a target’s gender contributes to the social framework of a moral dilemma, and thus the willingness to harm another. If this were the case, a target’s gender—and the social biases that associate males with strength and females with helplessness—may modulate the endorsement of harm, resulting in divergent altruistic behavior. In other words, these gender biases may influence females receiving greater chivalrous treatment (i.e., more protection from harm at the expense of self-gain) than their male counterparts. To test this, under both real and hypothetical contexts, and across different classes of moral dilemmas, we first explore whether a target’s gender influences the propensity to harm another.

We also wanted to investigate possible psychological mechanisms motivating an individual to harm another for self-gain. There is evidence that distinct patterns of moral judgments result from varying sensitivities to fairness and harm concerns (Haidt & Graham, 2007b). These two orientations are considered to be the most dominant foundations for moral decision-making, each capturing distinct perspectives: treat others fairly and help others in need (Gilligan & Attanucci, 1988). Theorists argue that individuals navigate moral challenges either by relying predominately on their sensitivity to harm and care considerations or through a well-developed calculus sensitive to justice and fairness concerns (Gilligan, 1982; Kohlberg & Hersh, 1977). A wealth of research now highlights that humans are highly attuned to both fairness (Fehr & Schmidt, 1999) and harm considerations (Cushman et al., 2012; Greene et al., 2009). Based on the literature, we were agnostic as to whether altruistic motivations would be better explained by the general tendency to endorse harm and care considerations or fairness and justice considerations. Thus, our second aim was to examine how a target’s gender and an individual’s considerations of harm and fairness interact to influence costly altruism—that is, helping or harming another at a cost to oneself.

To ask these questions, we probed behavior in two different types of moral dilemmas. First, we tested whether a target’s gender would have an effect on responses in the classic Trolley Dilemma (Foot, 1978; Thomson, 1976; Studies 1A and 1B), hypothesizing that if a target’s gender is instrumental for framing a moral scenario and shaping perceptions of harm, then even under hypothetical conditions where one must simulate the tensions, individuals should more readily agree to push a man—compared to a woman—in front of the trolley. In Study 2, we explored the effects of a target’s gender on moral behavior during real dyadic interactions between an intentional decider and a distressed target (Gray, Young, & Waytz, 2012). This dyadic paradigm—known as the Pain versus Gain (PvG) task (FeldmanHall et al., 2012)—requires participants to make decisions about how much money they would pay to reduce or prevent painful electric shocks from reaching a target (a confederate). This allowed us to test if participants exhibit differential patterns of altruistic behavior based on the target’s gender (i.e., paying more money to prevent harm from reaching a female compared to a male target). If gender and the accompanying biases—such as associating females with helplessness—contribute to the social framing of a moral dilemma, then participants who engage with a female target may display greater prosocial tendencies (i.e., more money paid out and less pain administered). Drawing on the classic work of Kohlberg and Gilligan (Gilligan, 1982; Kohlberg & Hersh, 1977) as well as the rich literature on moral trade-offs (Tetlock, Kristel, Elson, Green, & Lerner, 2000) and sacred values (Graham & Haidt, 2011), we further theorized that any observed differences in moral behavior would likely be a function of the interaction between a target’s gender and a participant’s individual trait differences in harm and fairness sensitivity. More specifically, increasing altruistic tendencies toward females may be related to how strongly participants identify with and value harm or fairness concepts.

## Studies 1A and 1B

### Participants

In Study 1A, 50 participants (20 females, mean age 32.5, *SD* ± 11.2) were recruited from the United States using the online labor market Amazon Mechanical Turk (AMT; Buhrmester, Kwang, & Gosling, 2011; Horton, Rand, & Zeckhauser, 2011; Mason & Suri, 2012; Paolacci, Chandler, & Ipeirotis, 2010). In Study 1B, 152 participants (78 females, mean age 37.1, *SD* ± 11.9) were recruited through AMT. Participants participated anonymously over the Internet and were not allowed to take part in more than one experimental session. All participants provided written informed consent, and the study was approved by Columbia University’s ethics committee.

## Method

Participants in Study 1A were presented with the classic variant of the Trolley Dilemma, the Footbridge Dilemma (Foot, 1978), and queried whether they would push a male or female bystander onto the tracks. Participants in Study 1B were randomly selected to read one of the three versions of the dilemma, where each vignette described a man, woman, or gender-neutral bystander on the bridge. The participant was then asked how willing they were to *“push the [man/woman/person] onto the path of the oncoming trolley,”* indicating on a 10-point analogue scale willingness to push (WTP). The aim was to determine whether there are observable gender biases during philosophical moral dilemmas, with the key variable being how readily a male or female bystander is pushed onto the tracks (i.e., harmed).

## Results and Discussion

**Figure 1.**
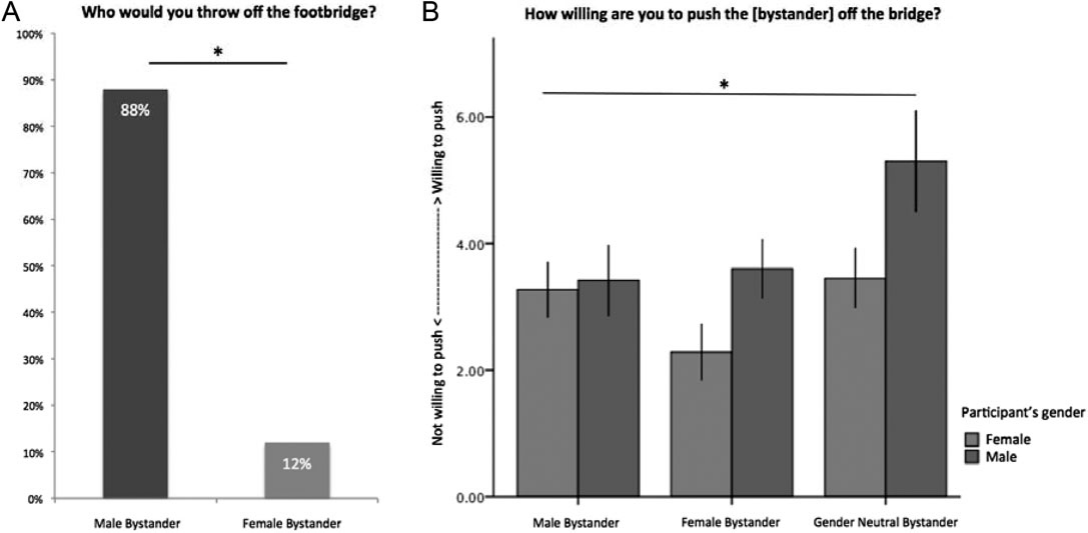
Behavior in footbridge dilemma. (A) When faced with either pushing a male or female bystander, participants overwhelmingly choose to sacrifice a male bystander. (B) A main effect of willingness to push was observed illustrating greatest willingness to push a bystander whose gender was not identified and least for a female bystander. A main effect of participant’s gender on willingness to push was also observed, with female participants less willing to push compared to male participants.

In Study 1A, 88% of participants reported that they would push the man off the footbridge (Pearson’s χ^2^ = 28.88, 1 *df*, *p* < .001, η^2^ = .57; Figure 1A), illustrating that participants significantly endorsed the preservation of a female over a male bystander’s welfare. Adding in participant’s gender as a factor revealed no significant effect (* p* > .6). During debriefing, participants suggested possible motivations for their responses explaining that “in a utilitarian situation, I value women and children over men” and “[pushing] a man is the moral thing to do, women are fragile and it would be morally wrong.”

In Study 1B, we manipulated gender and again explored participants’ WTP. We submitted WTP to a 3 (bystander gender) × 2 (participant gender) analysis of variance (ANOVA), where variables were coded as follows: −1 = female, 0 = unspecified gender, 1 = male. Results revealed a main effect of the bystander’s gender, *F*(2,146) = 3.8, *p* = .02, η^2^ = .05 (Figure 1B), such that participants were overall more willing to push a man (mean WTP = 3.3, *SD* ± 2.4, 10 = very willing) or a bystander with an unspecified gender (mean WTP = 4.3, *SD* ± 3.1) than a woman (mean WTP = 3.0, *SD* ± 2.4). We also observed a main effect of the participant’s gender, such that female participants were overall less willing to push (mean WTP = 3.2, *SD* ± 2.5) than male participants (mean WTP = 4.0, *SD* ± 2.9; *F*(1,146) = 6.4, *p* = .01, η^2^ = .04), which dovetails with previous research illustrating women are more sensitive to causing harm then men (Friesdorf, Conway, & Gawronski, 2015). The Bystander × Participant interaction did not survive significance (*p* > .1).

Together, this reveals that participants were less willing to push a woman than a man off the footbridge, suggesting that even within the hypothetical domain where the tensions are not as easily simulated (FeldmanHall et al., 2012), individuals take a target’s gender into account when contemplating harmful actions. We further observed that female participants were overall less willing to harm another, replicating previous work that not only are males sacrificed more often than females in classic trolley dilemmas but also that females are less likely to harm others (Eckel & Grossman, 2001; Skulmowski, Bunge, Kaspar, & Pipa, 2014).

## Study 2

### Participants

In Study 2, 57 adults were recruited from the UK Medical Research Council Cognition and Brain Sciences Unit volunteer panel (32 females; mean age 25.21, *SD* ± 4.83); sample size was based on previous similar work (FeldmanHall et al., 2012). In order to avoid priming moral attitudes and to minimize explicit moral reasoning during task performance, we recruited participants under the pretense of participating in an economic decision-making study. All participants provided written informed consent, and the study was approved by Cambridge University’s Psychology Research Ethics Committee. An independent group (*n* = 50; 24 males; mean age 36.1 years, *SD* ± 14.06) rated the attractiveness, approachability, and feelings toward both targets, finding that the male was significantly more attractive, approachable, and positive than the female target (all *p*s < .001, see Supplemental Material for details).

## Method

In the PvG task, participants (deciders) were presented with a series of 20 trials, each requiring a moral decision: benefit oneself financially or prevent harm to another. At the start of the experiment, deciders were given £20 and told that any money left at the end of the task would be multiplied up to 10-fold, giving them as much as £200. On each trial, £1 was at stake, and the choice was how much, if any, of the £1 to give up in order to prevent a painful but harmless electric shock from reaching the target on that trial. The more money paid out on a given trial, the lower the shock level inflicted on the target (index of costly altruism): Paying the full £1 would remove the shock altogether, while paying nothing would mean the target experienced the highest shock level on that trial. The key behavioral variable was how much money (£0–£20) deciders retained across the 20 trials, with larger amounts indicating that personal gain was prioritized over the target’s pain. Effectively, the more money the decider paid, the lower the shock level the target received on a given trial. Consequently, to stop all of the shocks across all 20 trials, deciders would need to spend all £20 (see Supplemental Material for full task details).

**Figure 2.**
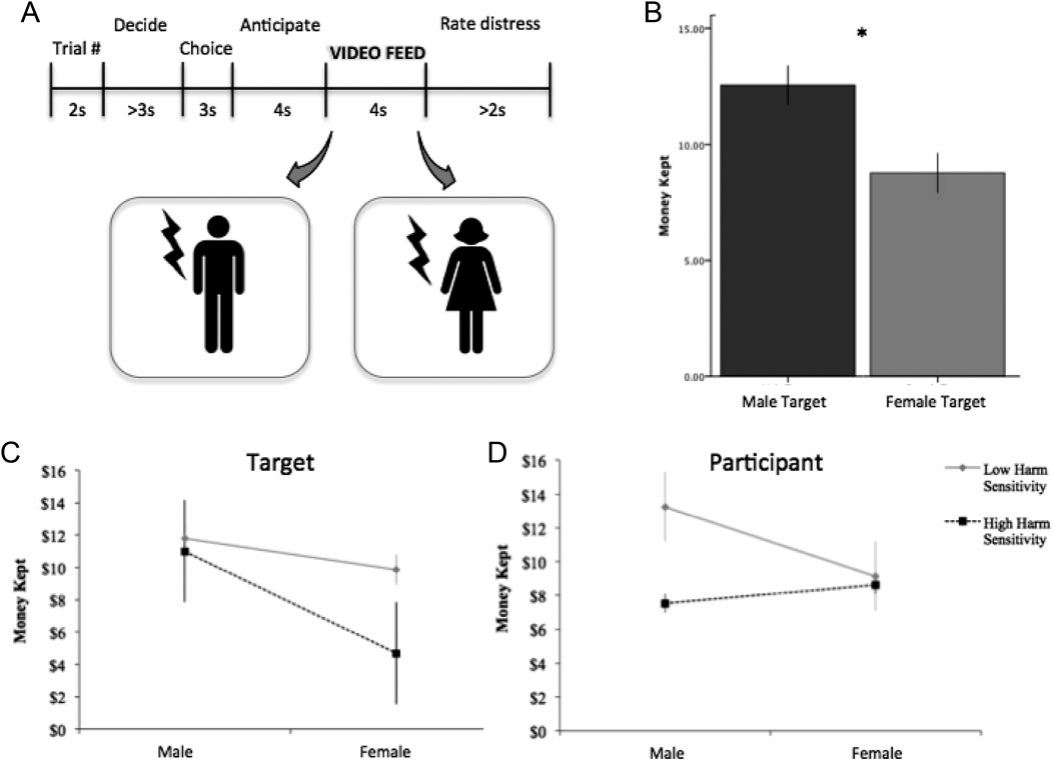
(A) Experimental design with schematic images of the two conditions—male and female targets—that participants observed during the video feed. (B) Participants kept significantly less money when interacting with a female target than a male target, *t*(55) = −3.16, *p* = .003. (C) The relationship between money kept (an index of selfish behavior) and the target’s gender as a function of trait harm sensitivity. (D) The relationship between money kept and a decider’s gender as a function of trait harm sensitivity. Variables were standardized before being entered into the regression. Regressions were graphed using the method of simple slopes (Aiken & West, 1991), where high harm sensitivity = 1 *SD* above the mean; low harm sensitivity = 1 *SD* below the mean. Error bars represent 1 *SE*.

Deciders were also required to view the administration of the shocks. This allowed us to manipulate the target’s gender by broadcasting a video of either a male target (Condition 1) or female target (Condition 2) responding to the shock (Figure 2A; we used this between-group design to control for the possibility of demand characteristics). Since the shocks were real, videos were prerated by an independent group to be matched across condition, such that both male and female targets elicited similar body and facial pain expressions that were directly yoked to the analogue scale presented to participants. To ensure that other potential factors besides a target’s gender were not driving behavior, we checked (using 8-point Likert-type scales in a subset of our participants during postexperimental questionnaires) that targets were matched on multiple dimensions including their familiarity, all independent *t*-tests: *t*(44) = −1.2, *p* = .234; similarity, *t*(44) = 0.403, *p* = .689; likeability, *t*(44) = −0.563, *p* = .577; and political orientations, *t*(44) = −0.007, *p* = .995.

### Moral Foundations Sacredness Scale (MFSS)

To explore potential moderating effects of harm and fairness considerations, we administered the MFSS (Graham & Haidt, 2011) after the PvG, which provides an index of the willingness to earn money at the expense of multiple moral considerations. The MFSS proposes that there are certain psychological foundations on which individuals build their moral systems and are organized along five dimensions (Graham & Haidt, 2011). Since the harm and fairness scales are believed to be most relevant to everyday life (Haidt & Graham, 2007a), and because there is a long-standing debate over which of these two dimensions best predicts moral behavior (Gilligan, 1982; Kohlberg & Hersh, 1977), we used these constructs as predictors of costly altruism. The scale measures how much money an individual is willing to receive to violate moral norms within each of the foundations (see Supplemental Material for more details), encapsulating whether or not a person is motivated (at the expense of money) to care for someone (harm) or is willing to immorally profit off others (fairness).

## Results

During the PvG task, deciders interacting with a female target kept significantly less money and thus gave significantly lower shocks (*n* = 34; £8.76/£20, *SD* ± 5.0) than deciders interacting with a male target, *n* = 23; £12.54/£20, *SD* ± 3.9; independent samples *t*-test: *t*(55) = −3.16, *p* = .003, Cohen’s *d* = .82; Figure 2B. This replicates the findings from Studies 1A and 1B in the real domain and under a different class of moral challenge, illustrating that harm endorsement is attenuated for female targets. Together, this suggests that a target’s gender can powerfully shift the perception of harm and lead to an increase in costly altruism (see Supplemental Material for further analysis including the influence of the decider’s gender on money kept as well as results of Study 4 replicating these findings in an online version of the PvG task).

Exploring deciders’ trait sensitivity to harm and fairness considerations (Graham & Haidt, 2011) revealed that female deciders reported significantly greater sensitivity to harm than male deciders, female mean harm sensitivity = 30.4, *SD* ± 5.1, male mean harm sensitivity = 26.2, *SD* ± 5.8; independent *t*-test: *t*(55) = −2.88, *p* = 0.02, Cohen’s *d* = .77. We did not observe a difference in deciders’ trait fairness levels, female mean fairness sensitivity = 25.0, *SD* ± 9.7, male mean fairness sensitivity = 27.1, *SD* ± 7.9; independent *t*-test: *t*(55) = 0.89, *p* = 0.38. To examine whether these individual trait differences in moral orientations moderate the relationship between altruism and a target’s and decider’s gender, we performed multiple regression analyses (Table 1). Money kept/shock delivered (index of costly altruism) was the dependent variable. The predictors at the first step (Model 1) were target’s gender, decider’s gender, and deciders’ individual trait harm scores (all *z*-scored). At the second step (Model 2), we entered each of the product terms of these variables, and at the third step (Model 3), we entered the three-way interaction term. Significant moderation is indicated by the fit of the model improving with each subsequent step (Aiken & West, 1991). We also ran this same regression with deciders’ fairness scores.

**Table 1.** Multiple Hierarchal Regression Study 2.

Variable	Model 1			Model 2			Model 3		
	*B*	*SE B*	β	*B*	*SE B*	β	*B*	*SE B*	β
Harm	−1.67	.61	−.34**	−1.55	.56	−.31**	−1.56	.57	−.36*
Decider gender (DG)	−0.76	.61	−.15	−0.75	.56	−.15	−0.74	.56	−.15
Target’s gender (TG)	−2.04	.57	−.41**	−1.87	.52	−.38**	−1.81	.58	−.36*
Harm × DG				1.31	.56	.25*	1.32	.57	.25*
Harm × TG				−1.16	.57	−.23*	−1.17	.58	−.24*
TG × DG				−0.46	.58	−.09	−0.48	.59	−.09
Harm × TG × DG							−0.16	.58	−.03
*R* ^2^		.31			.46			.46	
*F* for Δ*R* ^2^		8.03**			4.60*			0.07	

*p < .05. **p < .001.

We found a significant moderating role of harm sensitivity on both the effects of a target’s gender and a decider’s gender on altruistic behavior, Model 2: Δ*F*(3, 50) = 4.59, *p* = .006, Δ*r* ^2^ = .15, *r* ^2^ = total .46; Table 1. Figure 2C and 2D plots these significant interactions using the method of simple slopes (Aiken & West, 1991). We found that higher levels of trait harm sensitivity predicted greater altruism for female targets but not male targets (Figure 2C). That is, deciders high in harm sensitivity interacting with a female target kept significantly less money, thus preserving the female target’s physical welfare. We also found that trait levels of harm sensitivity played a moderating role on a decider’s gender and their subsequent choice to preserve the welfare of the target: Male deciders with low levels of trait harm sensitivity were significantly more selfish than males deciders with high levels of trait harm sensitivity (Figure 2D). This finding did not hold for female deciders, as females exhibited the same altruistic behavior regardless of their levels of trait harm sensitivity. Furthermore, there was no clear interaction relationship between an decider’s gender, a target’s gender, and trait harm sensitivity, Model 3: Δ*F*(1, 49) = 0.08, *p* = .78, Δ*r* ^2^ = .001, *r* ^2^ = total .46; Table 1.

While we found that fairness considerations influenced altruistic choice overall, Model 1: Δ*F*(3, 52) = 7.71, *p* < .001, Δ*r* ^2^ = .31, *r* ^2^ = total .27, we observed no evidence that fairness considerations had a moderating role on gender and altruistic choice, Model 2: Δ*F*(3, 49) = 2.1, *p* = .11, Δ*r* ^2^ = .08, *r* ^2^ = total .31. Together, these results demonstrate a strong relationship between a decider’s sensitivity to harm considerations and the target’s gender on altruistic choice as well as a strong relationship between a decider’s gender and their sensitivity to harm on altruistic choice.

## Studies 3A, 3B, and 3C

Given that we observed a target’s gender can bias one’s readiness to engage in harmful actions and that a decider’s considerations of harm—but not fairness concerns—modulate costly altruism, we next sought to explore possible motivations supporting this gender and harm interaction. One explanation is that females typically evoke more positive attitudes than males (Fazio & Olson, 2003) and are thus more likely to reap greater prosocial treatment. However, our post-task questionnaires probing attitudes toward the target significantly favored the male target, indicating that positive feelings for females are unlikely to be underlying the observed effect. An alternative explanation is rooted in the interaction between harm endorsement and adherence to societal norms, with the idea that it is more socially unacceptable to harm a female than a male (Becker & Wright, 2011; Crew, 1991; Viki, Abrams, & Hutchison, 2003; Wood & Eagly, 2002, 2009). There is also the possibility that individuals find it more emotionally aversive to harm a female, which in turn could enhance altruistic behavior in the PvG task (Cushman et al., 2012; Miller, Hannikainen, & Cushman, 2014; Pizarro, 2000). In the next studies, we probe whether these motivations might underlie an individual’s reluctance to harm a female target for monetary gain.

### Participants and Methods

A total of 352 participants were recruited for Studies 3A–3C, see Supplemental Material for details. Study 3A was a hypothetical analogue of the PvG task—where target’s gender was randomly manipulated to be female, male, or gender neutral (a between-subject design). Participants were queried about how much money most volunteers would keep and probed about societal perceptions of (1) harm, (2) pain tolerance, and (3) the chivalrous notion that men should protect women. Study 3B presented a subset of the same questions in Study 3A, except that questions pertained to both males and females (a within-subjects design). Study 3C randomly presented one of the three versions of the hypothetical PvG, and probed emotional aversion to the dilemma. See Supplemental Material for full list of questions and descriptive statistics from analyses in Studies 3A–3C; answers were recorded on 10-point Likert-type scales.

## Results

When probed about what other volunteers would do in the hypothetical analogue of the PvG, participants in Study 3A reported most volunteers would keep significantly less money when engaging with a female than a male or gender-neutral target, ANOVA: *F*(2, 148) = 3.8, *p* = .024, η^2^ = .05. Societal perceptions of pain tolerance revealed that women are believed to have a significantly lower pain tolerance than either men or a person whose gender was unspecified, ANOVA: *F*(2, 148) = 10.2, *p* < .001, η^2^ = .12. A similar pattern was observed regarding commonly held social norms that dictate how fair it is to harm a (man/woman/person); harming females was perceived as significantly more unfair than harming either a man or a gender-neutral person, ANOVA: *F*(2, 148) = 7.28, *p* = .001, η^2^ = .09.

**Figure 3.**
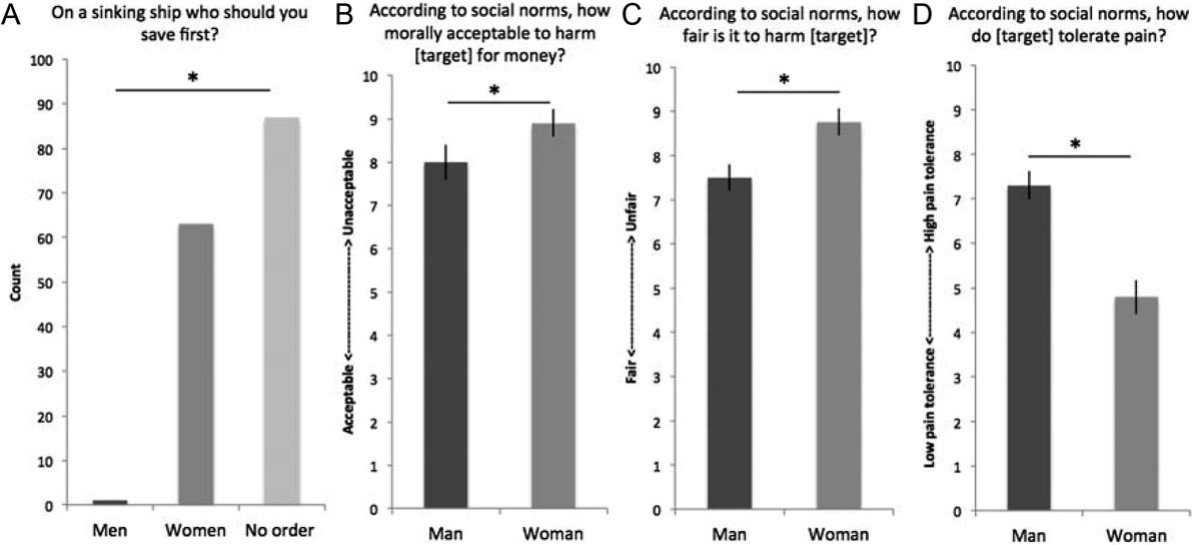
Societal norms motivating harm endorsement. (A) Participants reported there should be either no order for who is saved first on a sinking ship or that women should be saved first. (B) It is more unacceptable and (C) unfair to harm a woman than a man. (D) Men are perceived to have higher pain tolerances than women.

When queried about who should be saved first on a sinking ship, only one participant reported that men should be saved first (Pearson’s χ^2^ = 78.3, 2 *df*, *p* < .001, η^2^ = .52), and the rest of participants responded that there should either be no order or that women should be saved first (Figure 3A). Finally, participants reported that society generally subscribes to the chivalrous notion that men should lend more protection from harm to women than men, *t*(150) = −4.3, *p* < .001, Cohen’s *d* = .70.

Study 3B confirmed these findings in a within-subject design. Specifically, we observed that according to social norms, it is significantly (1) more morally unacceptable to harm a female for money, paired samples *t*-test: *t*(49) = −2.6, *p* = .01, Cohen’s *d* = .37: Figure 3B; (2) more unfair to harm a female, paired samples *t*-test: *t*(49) = −5.03, *p* < .001, Cohen’s *d* = .34: Figure 3C; and men have a significantly greater tolerance to pain, paired samples *t*-test: *t*(49) = 4.1, *p* < .001, Cohen’s *d* = .98: Figure 3D.

In Study 3C, we tested whether harm inflicted on males and females elicits different levels of emotional aversion. Participants responded to three questions relating to their own emotional aversion and level of emotional intensity after reading the hypothetical PvG scenario. Across conditions (male/female/gender neutral), we found no differences in the level of emotional aversion or level of emotional intensity (all *p*s > .1). That is, participants reported similar high levels of emotional aversion to reading about a male, female, and gender-neutral target in the PvG dilemma (see Supplemental Material for details).

Across these three studies, we investigate possible motivations supporting the finding that a target’s gender can bias an individual’s willingness to engage in harmful actions. The findings suggest that social norms regarding gender and harm considerations likely account for greater harming behavior toward a male than a female target. Moreover, there are widely held societal perceptions that females are less tolerant to pain, that it is unacceptable to harm females for personal gain, and that society endorses chivalrous behavior. Surprisingly, we found no differences in emotional aversion to reading about harming males versus females. These findings confirm perceptions of gender bias, and that these biases interact with harm considerations, helping to disambiguate why males are harmed more during the PvG task. While it is equally emotionally aversive to hurt any individual—regardless of their gender—that society perceives harming women as more morally unacceptable, suggests that gender bias and harm considerations play a large role in shaping moral action.

## General Discussion

Here we illustrate that across different types of moral dilemmas, and under both hypothetical and real contexts, moral choices to harm another are conditional on the social nature of the moral dyad and are thus relatively context dependent. Specifically, we show that moral behavior is modulated by the gender of the target individual, such that females are more readily protected from harm and are more often the recipients of costly altruism compared to their male counterparts. When asked to respond to a utilitarian-based moral dilemma, participants overwhelming responded that they would push a man—rather than a woman—in front of an oncoming trolley. When we tested this effect in a different class of moral challenge where self-benefit and another’s welfare are juxtaposed, we found that even under the same moral constraints where all components of the moral task were held constant, a target’s gender can shift an individual’s choice to be more or less altruistic, providing converging evidence that gender bias plays a significant role in moral behavior.

Research demonstrates that increasing the salience of harm has profound effects on moral behavior (Cushman et al., 2012; Greene et al., 2001), and the data presented here illustrate that the gender of the target is a critical feature that can shift the endorsement of harm and influence the altruistic response. One explanation for this is that beliefs about female and male characteristics bias how an individual perceives harming a target. Most people have widely shared beliefs and expectations about the traits and behaviors of males and females (i.e., Social Role Theory; Eagly, 1987). Males are typically associated with traits like aggression and dominance (Moss-Racusin et al., 2012; Sheltzer & Smith, 2014), and females are characteristically associated with traits such as nurturance and submission (Broverman, Vogel, Broverma, Clarkson, & Rosenkra, 1972; Cejka & Eagly, 1999; Rudman, Greenwald, & McGhee, 2001). These beliefs are easily and automatically activated (Banaji & Hardin, 1996b). For instance, individuals who were preconsciously primed with stereotypic characteristics like “sensitive” and “logical” (for female and male, respectively) were faster at identifying the gender of male or female names (Blair & Banaji, 1996). If females are canonically construed as sensitive and needing nurturance, then inflicting harm on a stereotypically weak target may be perceived as more salient and aversive than harming a target associated with strength and competence.

An alternative explanation suggests that people feel more positively about women than they do about men (Eagly, 1994). Our data, however, indicate that the male target was rated as more attractive, approachable, and positive compared to the female target. Thus, it seems unlikely that positive feelings for the female target influenced costly altruism. In fact, in light of the research linking attractiveness to increased helping (Benson, Karabenick, & Lerner, 1976), our findings that the female was helped more—despite being rated as less attractive and approachable—further supports and heightens the observed effect.

Regardless of an individual’s attractiveness and approachability, it is well documented that attitudes are shaped by implicit and explicit biases stemming from widespread cultural stereotypes about gender (Eagly, 1987, 1997; Eagly & Mladinic, 1989; Foschi, 2000; Goldin & Rouse, 2000; Nosek, Banaji, & Greenwald, 2002). Evidence of gender typical behaviors, such as females greater involvement in care-taking and communal behaviors, and males in more agentic and competitive behaviors (Gardner & Gabriel, 2004), illustrates that these attitudes can shape how males and females behave, including their engagement in prosocial behavior (Eagly, 2009). Although much more limited, some research has explored the other side of the social dyad; that is, whether gender stereotypes act as social norms influencing how targets are treated (Wood & Eagly, 2009). For example, women typically receive more help than their male counterparts (Eagly & Crowley, 1986), and in the Dictator game—where one can choose to forgo money in order to be fair—female players are allocated more money than male players (Saad & Gill, 2001). That we found that a target’s gender can also shape costly altruism, dovetails not only with male and female stereotypical characteristics, but also the notion that these gender biases have downstream effects on behavior. Indeed, we found there is a societally held notion that moral chivalry governs how morally unacceptable it is to harm a female. Social norms regarding pain tolerance and the notion that women should be protected from harm further confirmed that there is a societal belief that it is more morally unacceptable to harm a female than a male. Together, this suggests that these societal expectations about males and females and their relative gender role differences play a fundamental role in shaping the perceptions and framework of dyadic moral decisions.

We further observed that these behavioral patterns are moderated by the strength of the decider’s preference for harm—but not fairness—orientations, which suggests that concern for another’s well-being is a more salient motivator than concern for fairness. We also reveal that male deciders’ trait sensitivity to harm predicts altruistic behavior, while we found no support that female deciders’ sensitivity to harm motivates altruistic choice. Although at first glance this appears to counter the classic theory that females are more motivated by harm than their male counterparts (Gilligan, 1982; Gilligan & Attanucci, 1988), our data indicate that female deciders report overall higher harm sensitivity and have lower group variance than their male counterparts. Thus, it is possible that females may be more constant in their endorsement of harm considerations when navigating moral challenges, which is consistent with the broader theory that females are strongly motivated by harm and care orientations (Eagly, 1987; Eagly & Mladinic, 1989; Eagly & Wood, 1991; Gilligan & Attanucci, 1988). That male deciders’ altruistic behavior was predicted by their trait levels of harm sensitivity also fits with research illustrating that not only do males exhibit greater heterogeneity in delinquency involvement, but that they also engage in more antisocial behavior than females (Moffitt & Caspi, 2001).

Here we show that both hypothetical and real moral choices are influenced by the dyadic nature of the moral challenge. Even when all other social and contextual factors are held constant, a target’s gender can shift a decider’s choice to be more or less altruistic, suggesting that gender plays a significant role in how readily one violates the harm principle. In addition, these behavioral patterns are moderated by an individual’s sensitivity to harm but not fairness concerns. Together, these results illustrate that moral choices are not objectively implemented but instead are flexibly deployed relative to the individual’s moral orientations and the social context in which they are made.

## Supplementary Material

Supplementary material
