# Supplementary material for: Moral Chivalry: Gender and Harm Sensitivity Predict Costly Altruism
Source: Soc Psychol Personal Sci. 2016 May 25;7(6):542–51. doi: 10.1177/1948550616647448 (PMC4952565; doi:10.1177/1948550616647448)
Supplement: Supplementary material [file SPPS647448_suppl_mat.pdf]

SUPPLEMENTAL MATERIALS
Moral Chivalry: Gender and harm sensitivity predict costly altruism

STUDIES 1A & 1B

Additional Methods for Study 1A. Participants in Study 1A were presented with the classic variant of the Trolley dilemma, the Footbridge Dilemma (Foot, 1978), and queried with the following question about whether they would push a male or female bystander onto the tracks: *"A runaway trolley is hurtling down the tracks towards five people who will be killed if it proceeds on its present course. You are standing next to two people—a man and a woman of equal size—on a footbridge that spans the tracks. The only way to save the five people on the tracks is to push one of these people off the footbridge and into the path of the trolley. Do you choose to push the man or the woman on to the tracks?"*. Participants then had to key in either a 1 or 2 to indicate whom they would prefer to push onto the tracks. Participants were paid $.50 for participating in the study. The aim of Study 1 was to determine whether there are observable gender biases during hypothetical moral dilemmas, with the key variable being which gender is more often pushed onto the tracks (i.e., harmed).

Additional Methods for Study 1B. Participants in Study 1B were randomly selected to read one of three versions of the Footbridge Dilemma, where each vignette described either a man, woman or gender neutral bystander on the bridge. The participant was then queried as to whether they would *"push the [man/woman/person] off the footbridge and onto the path of the oncoming trolley",* indicating on a 10-point VAS how willing they were to push the bystander. After answering the moral vignette, participants completed the Social Dominance Orientation (SDO), which measures a predisposition to express social dominance under certain social conditions (i.e. preferences that endorse inequality in social groups). The SDO is also known to negatively correlate with altruism and empathy (Pratto et al., 1997). Participants were paid $.75 for participating in the study. The aim of Studies 1A and 1B were to determine whether there are observable gender biases during hypothetical moral dilemmas, with the key variable being how readily a male or female bystander is pushed onto the tracks (i.e., harmed).

Additional Results & Discussion. In Study 1B, we observed that participants with higher trait levels of SDO were significantly more willing to push the bystander off the footbridge, regardless of their gender (2 tailed Pearson's correlation, $r=.20$, $p=.02$). That one's willingness to push a bystander increased as trait levels of social dominance increased, indicates that the greater the dispositional desire to exert social dominance within society, the more willing they are to harm another.

STUDY 2

PvG Task Structure. The PvG task comprised a series of 8 screens per trial across 20 trials. Each trial began with a screen displaying the running amount of the Decider's bank total (£20 on Trial 1) and current trial number. Deciders then had to use a visual analogue scale (VAS) to select the amount of money they wanted to spend on that trial and thus the corresponding shock to be administered to the target. In other words, deciding how much money to spend effectively determined the shock administered to the target. This phase was partitioned into the "Decide" and "Select" periods. The Decide screen was presented for a fixed 3 seconds during which participants were asked to think about their decision. The Select screen was self-paced. After making a selection, Deciders saw a 3-second display of their choice before experiencing a 4-second anticipation phase during which Deciders were told their choice was being transmitted over the internal network to the adjacent testing lab where the target was connected to the shock generator. Following this anticipation period, Deciders viewed a 4 second video of the shock being administered to the target, or no shock if they had opted to spend the full £1 permitted on a given trial. Deciders believed they were viewing in real time via a video feed actual shocks being administered to a target sitting in a nearby testing laboratory. However, the videos were pre-recorded films, pre-rated by an independent group so as to be matched for shock level and corresponding pain intensity. Open-ended questions during debriefing revealed that Deciders believed the video feed, target, and shocks were genuine and in real time. Finally, agents used a 7-point VAS to rate their distress levels on viewing the consequences of their decision, before viewing a 4 second inter-trial-interval (ITI). At the conclusion of the 20 trials, Deciders were able to press a button that randomly multiplied any remaining money between 1 and 10 times, and this final amount was theirs to take home.

PvG Task Procedures. Each Decider (participants) and our two targets (male and female confederates) completed forms consenting to both receive and administer electric shocks. Both Deciders and targets were told that they were recruited from two different panels—one pre-selected to be the Decider (the true subject administering the shocks) and the other to be the Receiver (the confederate target receiving the shocks). During this brief period during which paperwork was filled out, the Decider and target were together and allowed to interact. Half way through reading the consent form, the target always repeated the same question about whether the "shock box" was safe and the experimenter responded that the device was approved for clinical use within the laboratory. Both the Decider and target were then taken to the testing laboratory housing the electric shock generator, a Digitimer DS7A, and briefed on the set-up of the experiment. The Decider, sitting in the place where the target would subsequently sit for the duration of the experiment, received the low-level shock choice and was asked to rate his/her own pain on a 10-point scale. This was to provide the Decider with explicit information concerning what the target would later experience during the PvG task. The Decider was then taken to another room while the target was connected to the shock generator. Once there, the Decider was endowed with a £20 note and told that the money could be used to stop or attenuate the shocks planned for the target.

Experimenter Script during Consenting Participants for PvG. Both experimenters greet participants in reception. The target waits until the true subject arrives and is given their volunteer badge. Once this happens, the target enters the waiting room. The experimenter then says: *"Thank you both for coming to the CBU as you are both aware from our correspondence over the phone of via email, this experiment is regarding economic decision making. It also involves the administration of shocks. Which one of you is {target's name]? Great, as you know, you have been recruited to be the participant who is receiving shocks via our volunteer panel. You must be [Participant's Name], you have been recruited to make decisions regarding money and shocks via the Graduate bulletin. I will explain both tasks in detail once we move down to the testing facility, but for now will you both take a minute to read over and sign these consent forms?"*

Post-Experimental Questionnaires. After the experimental session was finished, Deciders answered a series of questions that asked them to indicate on an 8-point analog scales (ranging

from 1 to 8): 1) whether they felt they were being watched during the experiment, 2) how much respective responsibility they, the experimenter, and the target had for the electric stimulations administered, 3) and whether there was any doubt as to the veracity of the paradigm. Choices could not be explained by Deciders modifying their decisions in response to reputation management or feelings of being watched (Landsberger, 1958) as we found no correlation between Deciders' ratings of beliefs about being watched and amount of money kept ($r=-.10$, $p=.52$, 2-tailed). Furthermore, Deciders rated themselves significantly more responsible for their actions than either the Experimenter or the target (mean responsibility for self 5.96, SD±2.19; mean responsibility for Experimenter 4.12, SD±2.35; mean responsibility for target 4.74, SD±2.46; all $Ps<0.005$). Finally, results reveal there was no significant correlation between Deciders' ratings of the believability of the task and their behavioral performance (shock delivered/money kept), (Pearson's correlation; $r=-.16$, $p=0.25$, 2-tailed).

Attractiveness & Approachability Ratings. An independent group (N=50; 24 males; mean age 36.1 years, SD±14.06) was recruited from AMT and asked to rate the attractiveness, approachability, and feelings towards both targets. Results reveal that the male target was rated as significantly more attractive (mean 5.42, SD±1.9) compared to the female target (mean 4.12, SD±1.9; paired t-test: $t(49)=4.79$, $p<0.001$ Cohen's $d=.68$), and significantly more approachable (mean 3.80, SD±2.2) than the female target (mean 4.12, SD±1.9; $t(49)=6.16$, $p<0.001$ Cohen's $d=.16$). Participants also reported feeling significantly more positive about the male target (mean 5.52, SD±1.7) compared to the female target (mean 4.2, SD±1.8; $t(49)=4.6$, $p<0.001$ Cohen's $d=.75$). We found no evidence that these ratings were biased by participants' gender (i.e. male and female participants rated the male target similarly; all $Ps>0.1$ for both targets' ratings), or that male participants (N=24) found the female target (mean 3.91, SD±1.9) more attractive than the male target (mean 5.00, SD±1.9, $p>0.07$).

Moral Foundations Sacredness Scale. These foundations within the MFSS are organized along five dimensions: harm, fairness, in-group, authority, and purity. The scale measures how much money an individual is willing to receive to violate moral norms within each of the five foundations. For example, a prototypical question concerning harm asks how much on a scale of *"I would do it for free"* ($0) to *"never for any amount of money"* (with a scale increasing by the

power of ten, \$10 to \$1 million) would you be willing to *"kick a dog in the head, hard"*. An example of a question on the fairness scale asks if an individual would be willing to *"cheat in a game of cards played for money with some people you don't know very well"*. These item examples encapsulate whether or not a person is motivated (at the expense of money) to care for someone vulnerable (harm), or is willing to immorally profit off others (fairness). In short, this scale provides a good measure of how willing people perceive themselves to make money at the expense of another's harm or fairness considerations.

Results. A 2x2 ANOVA exploring the effects of target gender and Decider gender on money kept revealed that Deciders interacting with a female target kept significantly less money and thus gave significantly lower shocks (£8.76/£20, SD±5.0) than Deciders interacting with a male target (£12.54/£20 SD±3.9; $F(1,53)=9.5$, $p=0.003$, $\eta^2=.15$, Fig 2B), and a marginally significant effect of an Decider's gender on money kept such that female Deciders kept less money overall in the PvG task (male Decider mean £11.48/£20 SD±4.7, female Decider mean £9.23/£20 SD±4.9: ANOVA, $F(1,53)=3.4$, $p=0.09$, $\eta^2=.06$, Fig S1). There was no interaction between Decider and target gender ($p>.1$). Interestingly, the least amount of money kept (most prosocial choice) was when female Deciders interacted with female targets (\$6.87 SD±4.1), mirroring the findings observed in Study 1B. These results indicate that females are more sensitive to causing harm than males, dovetailing with previous work (Friesdorf, 2015).

Because the male target was perceived as more attractive and approachable compared to the female target, it is possible that participants in the PvG gave higher shocks and kept more money with male target because they believed that he would be more agreeable to such treatment. While this is a possibility, it is unlikely to explain the results presented in the manuscript for a number of reasons. First, we illustrate this effect over multiple additional studies in different classes of moral dilemmas where this issue is not a confound. Second, in none of the extensive debriefing sessions did participants allude to such a strategy.

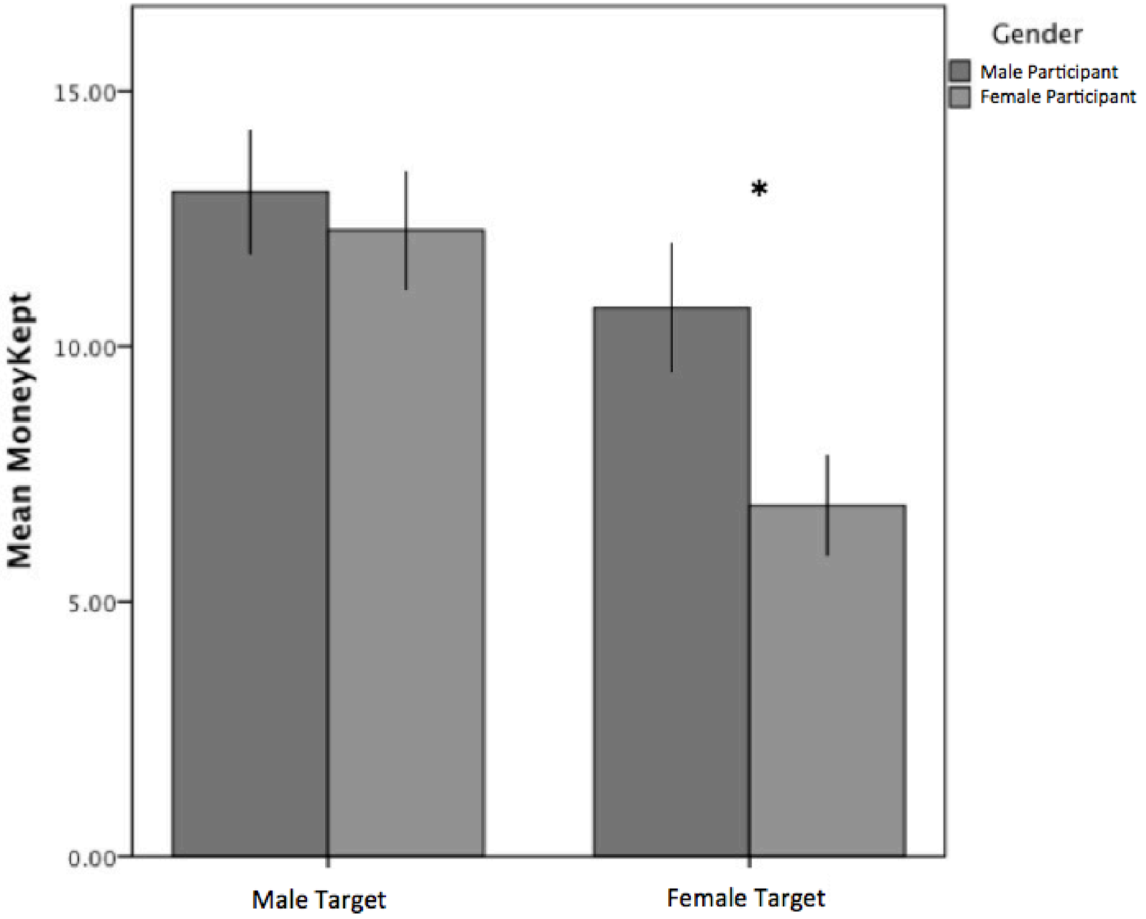

Fig S1 | *Behavioral Data from study 2 broken out into participant gender and target gender. Error bars reflect 1 SEM.*

STUDY 3A

Participants. Using AMT, we recruited 151 participants for Study 3A (65 females, mean age 34.5, SD±11.3), 50 participants for Study 3B (21 females, mean age 34.3, SD±11.2), and 151 participants for Study 3C (88 females, mean age 38.3, SD±13.0). Participants participated anonymously over the Internet and were not allowed to take part in more than one experimental session. All participants provided written informed consent, and the study was approved by Columbia University's ethics committee.

Using a between subject design, participants were presented with the following written dilemma: *"Two volunteers have agreed to participate in a psychology experiment. One volunteer is given $20 at the beginning of the experiment and is told that any remaining money at the end has the possibility of being multiplied up to ten times; they will be able to keep this money. To*

participate in the experiment their task is to decide whether the other volunteer—[John/Sarah/other volunteer]—will receive a series of painful electric shocks. It is explained that they can use the money they were given to reduce the pain of the shocks or to stop the shocks from reaching [John/Sarah/other volunteer]. The volunteer decides to keep $17 of the $20, thus applying a number of painful (but harmless) electric shocks to [John/Sarah/other volunteer]." Participants were then asked to respond to the following questions using a 10-point likert scale:

1. How much money do you think most volunteers would keep in this situation? ($0-$20).
2. How morally acceptable is it to harm a [man/woman/person] for money according to general societal norms?
3. Accordingly the general perceptions of society, how well do [men/women/people] tolerate pain?
4. Accordingly the general perceptions of society, do you think it fair to harm a [man/woman/person]?
5. Consider a sinking ship, who would you save first
    a. The men
    b. The women
    c. There should not be order for who is saved first
6. Do you think that society generally believes the notion that men should lend more protection from harm to women than to men?

Supplemental Results Study 3A. When probed about what other volunteers would do in the hypothetical analogue of the PvG, participants in Study 3A reported that most volunteers would keep significantly less money when engaging with a female (mean money kept $8.13, SD±5.6) than a male (mean $9.42, SD±5.4) or gender neutral target (mean $11.30 SD±6.2; Q1, ANOVA: $F(2,148)=3.8$, p=.024, $\eta^2=.05$). That is, participants assumed other volunteers would preserve the female target's welfare more so than the male or gender-neutral target's welfare. We did not observe any differences between perceived moral acceptability of harming males and females (Q2, p>.1), although this could be due to the fact that responses were near ceiling. When queried about societal perceptions of pain tolerance amongst males and females, participants reported that females are believed to have a significantly lower tolerance to pain (mean pain tolerance 5.5, SD±2.4) than either men (mean 7.4, SD±2.2) or a person whose gender was unspecified (mean 7.3, SD±2.3; Q3, ANOVA: $F(2,148)=10.2$, p<.001, $\eta^2=.12$). A similar pattern was observed regarding societal norms dictating how fair it is to harm a [man/woman/person]; Harming females was perceived as significantly more unfair (mean 9.0, SD±1.8) than harming either a man (mean 7.9, SD±2.0) or a gender neutral person (mean 7.5 SD±2.1; Q4, ANOVA: $F(2,148)=7.28$, p=.001, $\eta^2=.09$).

When queried about who should be saved first on a sinking ship, only one participant reported that men should be saved first (Q5, Pearson's X^2=78.3, 2df, p<0.001 η^2=.52), and the majority of participants responded that there should either be no order or that women should be saved first (Fig 3A). Finally, participants reported that society generally subscribes to the chivalrous notion that men should lend more protection from harm to women (mean agreement 4.1, SD±2.3 out of 10 where 1=totally agree, confirmed in a one sample t-test against a test value of 5=neutral; Q6, t(150)=-4.3, p<0.001 Cohen's *d*=.70).

STUDY 3B

Using a within subject design (subjects received both a male and female version), participants were presented with the hypothetical analogue of the PvG (described above in 3A). Participants were then asked to respond to the following questions using a 10-point likert scale:

1. How morally acceptable is it to harm a [man/woman/person] for money according to general societal norms?
2. Accordingly the general perceptions of society, how well do [men/women/people] tolerate pain?
3. Accordingly the general perceptions of society, do you think it fair to harm a [man/woman/person]?

STUDY 3C

Using a between subject design, participants were presented with above hypothetical analogue of the PvG (described above). Participants were then asked to respond to the following questions using a 10-point likert scale:

1. How emotionally aversive do you find it that [John/Sarah/the second volunteer] is harmed for money?
2. How emotionally intense was it for you to read this scenario and imagine [John/Sarah/the second volunteer] being harmed for money?
3. How emotionally intense would it be for you to harm [John/Sarah/the second volunteer] for money?

STUDY 4

Participants. 120 adults were recruited from the United States using AMT (Mason & Suri, 2012). 10 subjects were removed for completing the study two quickly or failing comprehension checks at the end of the task. The remaining participants, N=110 (42 females; mean age 32.6 years, SD±10.34) were included in the analysis. Participants participated anonymously over the

Internet and were not allowed to participate in more than one experimental session. All participants provided written informed consent, and the study was approved by the Columbia University's ethics committee.

Methods. In the online version of the PvG, participants were presented with a series of 20 trials, each of which involved a hypothetical moral decision that pitted maximizing their own payout against preventing a series of electric shocks aimed at a target individual. The online PvG followed the same structure as the laboratory version with some key differences: on each trial, participants could only earn $.10 by indicating that they would like to administer a hypothetical shock to a target subject. Instead of a video feed of a target individual receiving electric shocks, participants either engaged with a image of a male target or female target hooked up to the Digitimer (images were still shots taken from the videos of the target individuals presented in Study 2). After the PvG, participants completed the MFSS and Ambivalent Sexism Inventory (ASI) (Glick & Fiske, 1996). The MFSS was administered in order to replicate our findings that harm sensitivity predicts altruistic behavior as observed in Study 2. The ASI was administered in order to explore whether hostile sexism or benevolent sexism would be a better predictor the gender effects in the Pain versus Gain task. Overall, the ASI predicts ambivalent attitudes towards women. The two subscales correlate with negative attitudes towards women (hostile) and positive attitudes towards women (benevolent). Participants were paid $.75 for participating in the study and could make up to $2.00 depending on their choices during the task.

Results. Results reveal a similar pattern of findings of gender bias influencing perceptions of harm observed in Studies 1 and 2. In Study 4, Deciders deciding to shock a male target kept significantly more money (N=51: $1.86/$2.00) compared to those deciding to shock a female target (N=59: $1.63/$2.00; ANOVA $F(1,108)=5.34$, $p=.023$, $\eta^2=.05$). These results confirm that the perception of harm is influenced by a target's gender, such that individuals respond more altruistically when engaging with a female target compared with a male target.

First, we did not find any significant results for the Ambivalent Sexism Inventory (or the two subscales: benevolent/hostile) influencing the altruistic response (all $Ps>0.5$), or moderating gender differences in the PvG task. Second, the fact that we didn't replicate the interaction between harm sensitivity and a target's gender may be because there were a number of key

differences between Studies 2 and 4. First, Study 2 was run in the laboratory and required subjects to make real decisions about money they could take home and shocks that were being administered to a participant they had met and spent some time with. Study 4 was run online via Amazon Mechanical Turk, and thus, participants were asked to imagine the shocks being administered. Moreover, the participants in Study 4 never met the target individual and only viewed a still image of the target individual hooked up to the Digitimer. Previous work from our own lab has shown that when the moral dilemma is sufficiently stripped of social cues and context—and an individual must simulate many of the tensions of the moral dilemma—responses do not reliably parallel real behavior (FeldmanHall et al., 2012).

As in Study 2, in Study 4 female Deciders reported significantly greater sensitivity to harm than male Deciders (female mean harm sensitivity 31.2, SD±4.1, male mean harm sensitivity 28.2, SD±5.8; independent t-test: $t(108)=-2.9$, $p=0.005$). We again did not observe a difference in Deciders' trait fairness levels (female mean fairness sensitivity 29.9, SD±4.1, male mean fairness sensitivity 28.4, SD±6.9; independent t-test: $t(108)=-1.3$, $p=0.20$). To replicate that harm sensitivity plays a role in costly altruism—we performed multiple regression analyses using the same three models described in Study 2. We again found significant main effects of both a target's gender: participants spent more money preserving a female target—and, harm sensitivity on altruistic behavior—such that increasing sensitivity to harm predicted greater altruism (Table S2, Model 1: $F(3, 106)=5.71$, $p=0.001$, r^2=total 0.14). We did not find any significant result for the interactive effect with harm and gender (Table S2, Model 2), as we had in Study 2.

However, that the main effects of gender on altruistic choice were replicated within the hypothetical domain (endorsing harm more for a male than a female), suggests that gender bias and its influence on harm perception is so robust that it is insensitive to changes in social context, including class of moral dilemma (i.e., utilitarian versus self-benefit). Considering that we were unable to replicate the finding that the relationship between gender and altruism is moderated by an individual's sensitivity to harm in the hypothetical domain, suggests that this moderating effect is more subtle, and is sensitive to the demands of the moral dilemma. It is plausible that the relationship between harm sensitivity and a target's gender moderating costly altruism observed in Study 2 requires that the individual observe real harm—as opposed to

simulated harm. Future work can further decompose how these individual differences differentially contribute to harm perception during real and hypothetical moral dilemmas.

Table S2: Multiple Hierarchal Regression Study 4

	Model 1			Model 2			Model 3		
Variable	*B*	*SE B*	β	*B*	*SE B*	β	*B*	*SE B*	β
Harm	-.155	.05	-.29*	-.168	.05	.32*	-.167	.06	.32*
Agent's Gender (AG)	-.017	.05	-.25	-.011	.05	-.02	-.012	.05	-.02
Target's Gender (TG)	-.131	.05	-.25*	-.14	.05	-.26*	-.13	.05	-.26*
Harm x AG				-.05	.06	.10	-.05	.06	.08
Harm x TG				-.07	.05	-.12	-.07	.06	-.13
TG x AG				-.009	.05	-.02	-.007	.05	-.01
Harm x TG x AG							-.01	.06	-.02
R^2		.14			.16			.16	
F for ΔR^2		5.71**			0.95			.03	

*P<0.05, **P<0.001

SUPPLEMENTAL REFERENCES

FeldmanHall, O., Mobbs, D., Evans, D., Hiscox, L., Navardy, L. & Dalgleish, T. (2012). What we say and what we do: The relationship between real and hypothetical moral choices. *Cognition*.

Foot, P. (1978). *The Problem of Abortion and the Doctrine of the Double Effect in Virtues and Vices* Oxford: Basil Blackwell.

Friesdorf, R., Conway, P. & Gawronski, B. (2015). Gender differences in responses to moral dilemmas: a process dissociation analysis. *Pers Soc Psychol Bull,* 41**,** 696-713.

Glick, P. & Fiske, S. T. (1996). The Ambivalent Sexism Inventory: Differentiating hostile and benevolent sexism. *Journal of Personality and Social Psychology,* 70**,** 491-512.

Landsberger, H. (1958). *Hawthorne Revisited: Management and the Worker, Its Critics, and Developments in Human Relations in Industry,* Ithaca, New York, Distribution Center, N.Y.S. School of Industrial and Labor Relations, Cornell University.

Mason, W. & Suri, S. (2012). Conducting behavioral research on Amazon's Mechanical Turk. *Behav Res Methods,* 44**,** 1-23.

Pratto, F., Stallworth, L. M. & Sidanius, J. (1997). The gender gap: Differences in political attitudes and social dominance orientation. *British Journal of Social Psychology,* 36**,** 49-68.
